# *Toxoplasma gondii* in Wild Cervids and Wild Canids: Feeding Ecology, Environmental Exposure, and Trophic Transmission

**DOI:** 10.3390/pathogens15070746

**Published:** 2026-07-16

**Authors:** Vy Dinh Bao Tran, Dong-Hyuk Jeong

**Affiliations:** 1 Laboratory of Wildlife and Conservation Medicine, College of Veterinary Medicine, Chungbuk National University, Cheongju 28644, Republic of Korea; vybao@chungbuk.ac.kr; 2 Wildlife Center of Chungbuk, Cheongju 28116, Republic of Korea

**Keywords:** *Toxoplasma gondii*, wild cervids, wild canids, disease ecology, trophic transmission, environmental contamination, wildlife surveillance, One Health

## Abstract

*Toxoplasma gondii* is a zoonotic protozoan transmitted by environmentally persistent oocysts and by tissue cysts in infected prey or meat. Although wild cervids and wild canids are increasingly used as wildlife sentinels, their complementary ecological roles in integrated *T. gondii* surveillance have not been comprehensively synthesized. This structured narrative review compares infection evidence in five wild cervid species and three wild canid species to examine how feeding ecology shapes exposure and to assess their complementary value in wildlife surveillance. Peer-reviewed literature published between 2000 and 2026 was retrieved from PubMed, Scopus, ScienceDirect, and Google Scholar. Studies reporting evidence of *T. gondii* exposure or infection in wild cervids or wild canids were included, with serological evidence evaluated separately from molecular or histological detection. Cervids showed geographically variable exposure consistent with ingestion of oocysts from contaminated vegetation, soil, and water, supporting their use as sentinels of environmental contamination. Wild canids often showed higher reported seropositivity, although direct comparisons were limited by assay, sampling, and demographic heterogeneity. Their predatory, scavenging, and omnivorous diets allow access to both environmental oocysts and tissue cysts. Cervids and canids should therefore be treated as complementary rather than interchangeable indicators: cervids primarily reflect environmental exposure, whereas canids integrate environmental and trophic transmission. This review provides an ecological framework to support integrated One Health wildlife surveillance by combining environmental and trophic indicators for improved risk assessment and food-safety planning.

## 1. Introduction

**Figure 1:**
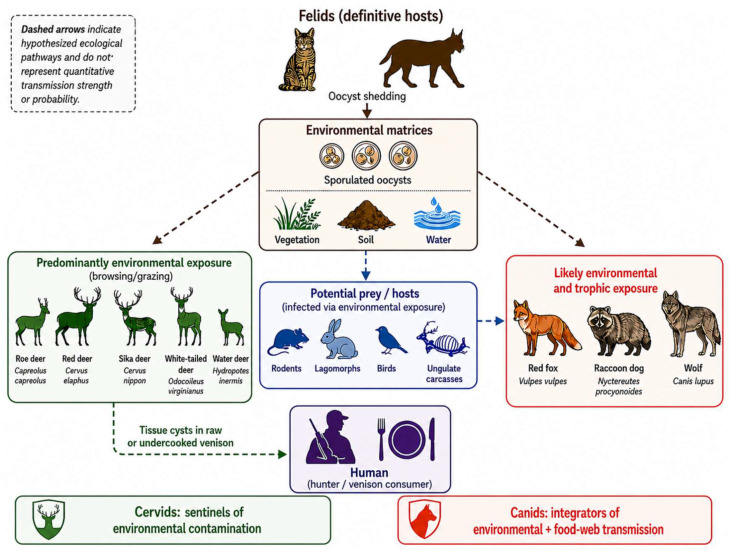
Conceptual framework for complementary surveillance of *Toxoplasma gondii* in wild cervids and wild canids. Felids introduce oocysts into terrestrial environments; cervids primarily acquire infection through contaminated vegetation, soil, and water, whereas canids can acquire infection through both environmental exposure and consumption of tissue cysts in prey or carrion. Cervid meat creates a separate foodborne pathway to hunters and consumers. Arrows depict conceptual transmission routes and do not encode transmission strength or probability. Solid arrows denote established biological routes, whereas dashed arrows indicate inferred ecological or surveillance relationships.

*Toxoplasma gondii* is a globally distributed, obligate intracellular protozoan capable of infecting virtually all warm-blooded vertebrates. Felids are the definitive hosts and can shed environmentally resistant oocysts, whereas mammals and birds generally act as intermediate hosts in which persistent tissue cysts develop. Infection is acquired principally by ingestion of sporulated oocysts in contaminated water, soil, or food; ingestion of tissue cysts in prey or meat; or transplacental transmission. These parallel routes connect wildlife, domestic animals, environmental matrices, and humans within a single epidemiological system [1,2,3]. A conceptual overview of the environmental and trophic transmission pathways of *T. gondii* and the respective roles of cervids and canids in wildlife surveillance is presented in Figure 1.

Wildlife epidemiology is particularly informative for *T. gondii* because host species differ markedly in trophic level, movement, habitat use, longevity, and contact with human-modified environments. A recent global macroecological analysis of 485 studies covering 533 free-ranging mammalian species showed that ecological traits, particularly carnivory, aquatic habitat use, and dispersal distance, were associated with infection probability. That analysis provides broad support for the importance of trophic and environmental pathways, but taxon-focused comparisons are still needed to translate macroecological patterns into practical surveillance designs [4]. Recent meta-analyses estimated an apparent pooled prevalence of 12% in wild ruminants and 16.6% in wild birds, demonstrating substantial but heterogeneous exposure across wildlife taxa and reinforcing the relevance of wildlife surveillance for environmental and foodborne risk assessment [5,6].

Wild cervids are obligate herbivores that feed on grasses, forbs, shrubs, and browse, depending on species and habitat. Species such as red deer (*Cervus elaphus*), roe deer (*Capreolus capreolus*), sika deer (*Cervus nippon*), white-tailed deer (*Odocoileus virginianus*), and Korean water deer (*Hydropotes inermis*) have been frequently investigated as wildlife hosts of *T. gondii*. Because they acquire infection primarily through ingestion of oocysts contaminating vegetation, soil, and water rather than through prey consumption, cervid infection generally reflects environmental exposure. In addition, these species are harvested for game meat in many regions, making *T. gondii* infection relevant to food safety because viable tissue cysts may occur in skeletal muscle and other edible tissues [7,8,9].

Wild canids occupy a contrasting ecological position. Red foxes (*Vulpes vulpes*), raccoon dogs (*Nyctereutes procyonoides*), and wolves (*Canis lupus*) consume vertebrate prey, carrion, invertebrates, and, depending on species and habitat, plant material and anthropogenic food. They may therefore encounter both environmental oocysts and tissue cysts. Infection in canids can integrate exposure across prey communities and space, making these hosts potential sentinels of food-web circulation rather than simple indicators of a single route [10,11].

Previous wildlife reviews have often covered very broad taxonomic assemblages or focused on one host group. This review addresses a narrower ecological comparison between free-ranging wild cervids and wild canids. The review specifically considers five cervid species (red deer, roe deer, sika deer, white-tailed deer, and Korean water deer) and three canid species (red fox, raccoon dog, and wolf). Species were selected a priori based on four criteria: (i) availability of extractable prevalence data from free-ranging populations; (ii) representation of contrasting herbivorous and predatory/scavenging feeding guilds to facilitate ecological comparisons of *T. gondii* transmission pathways; (iii) broad geographic distribution and epidemiological relevance of the selected host species; and (iv) their importance for wildlife surveillance, game meat exposure, and terrestrial ecosystem monitoring. The literature search was not restricted by geographic region; studies from all regions were considered. However, the eligible studies identified through the predefined inclusion criteria were derived primarily from Europe, Asia, and North America, and these therefore constituted the basis of the comparative analysis. The aims were to: (i) summarize reported serological and direct-detection evidence in selected species; (ii) evaluate how feeding ecology and trophic position shape plausible transmission pathways; (iii) identify diagnostic and sampling limitations that constrain cross-study comparisons; and (iv) propose a practical framework for complementary cervid–canid surveillance, with particular attention to East Asian systems and game-meat risk. The review does not assume that between-species differences in reported positivity are caused by diet alone; rather, it treats feeding ecology as one component of a broader exposure system that also includes diagnostic method, host age, climate, landscape context, and felid-associated contamination.

## 2. Review Approach

### 2.1. Search Strategy and Eligibility Criteria

A structured narrative review was conducted to preserve ecological and methodological detail while avoiding inappropriate pooled estimates across heterogeneous studies. Peer-reviewed literature published from 2000 to 2026 was retrieved from PubMed, Scopus, ScienceDirect, and Google Scholar. This period was selected to focus on contemporary wildlife surveillance studies using modern serological and molecular diagnostic approaches. Studies reporting evidence of *Toxoplasma gondii* exposure or infection in wild cervids or wild canids were included in the review. Search strings combined terms for the pathogen, host groups, epidemiology, and transmission, including: (“*Toxoplasma gondii*” OR toxoplasmosis) AND (wild cervid OR deer OR “roe deer” OR “*Capreolus capreolus*” OR “red deer” OR “*Cervus elaphus*” OR “sika deer” OR “*Cervus nippon*” OR “white-tailed deer” OR “*Odocoileus virginianus*” OR “water deer” OR “*Hydropotes inermis*” OR wild canid OR fox OR “*Canis lupus*” OR wolf OR “*Vulpes vulpes*” OR “raccoon dog” OR “*Nyctereutes procyonoides*”) AND (prevalence OR seroprevalence OR epidemiology OR transmission OR serology OR PCR OR isolation OR predation OR scavenging). Reference lists of relevant articles and reviews were screened manually to identify additional studies.

Studies were eligible when they: (i) examined naturally occurring *T. gondii* infection or exposure in free-ranging cervids or wild canids; (ii) reported a diagnostic method and a sample denominator or otherwise provided interpretable prevalence information; and (iii) were available in English as peer-reviewed articles. Captive-only studies, experimental infections, studies confined to domestic dogs or farmed cervids, records without host-specific results, and non-peer-reviewed reports were excluded from the evidence tables. Broad reviews, diagnostic-method papers, and ecological studies were retained for contextual interpretation but were not treated as prevalence records. Because the review was designed as a structured narrative synthesis rather than a systematic review or meta-analysis, no pooled prevalence or formal risk-of-bias score was calculated. The included studies varied considerably in host species, geographic location, diagnostic methods, sample types, and study design, limiting the validity of direct quantitative comparisons across studies. Consequently, the evidence was synthesized by examining recurring ecological patterns while interpreting reported findings in the context of methodological differences, rather than by combining prevalence estimates across heterogeneous datasets.

### 2.2. Data Extraction and Evidence Classification

**Figure 2:**
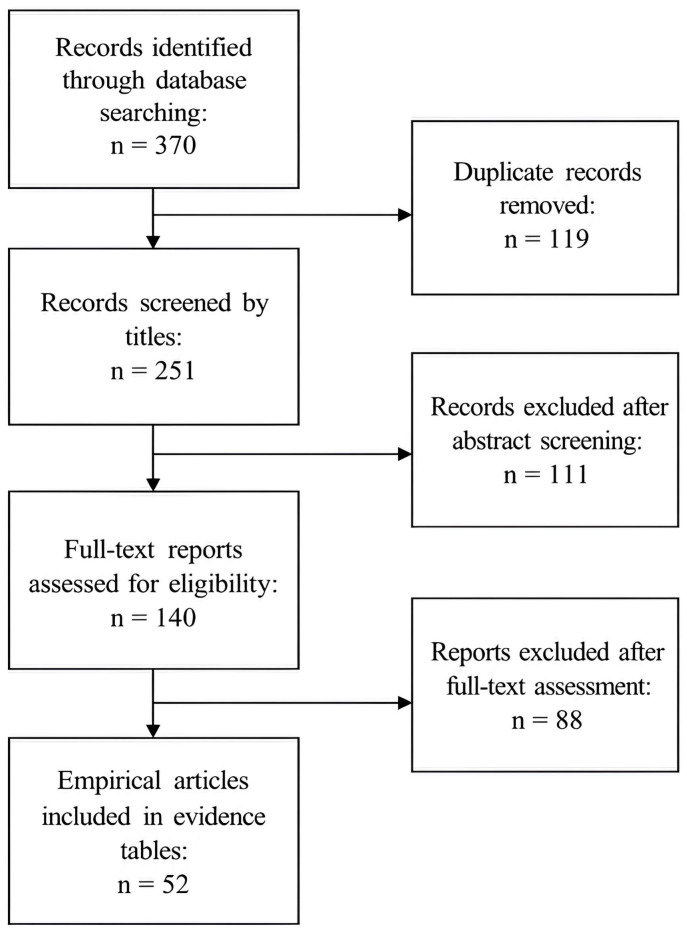
Flow diagram of the study selection process.

For each prevalence record, the host species, continent, country, diagnostic method, number positive and tested when available, and reported percentage were extracted. The database search identified 370 records. After the removal of 119 duplicate records, 251 unique records were screened by title and abstract. Of these, 111 records were excluded as outside the review scope, and 140 full-text reports were assessed for eligibility. After full-text assessment, 88 reports were excluded because they did not meet the predefined eligibility criteria, resulting in 52 empirical articles included in the evidence tables. A summary of the selection process is provided in Figure 2. Multiple methods applied to the same host population were retained as separate records because they address different biological endpoints. Reported values were checked for arithmetic consistency; when a source table provided only a percentage and total sample size, the numerator was not reconstructed unless it was stated elsewhere in the article.

Diagnostic results were divided into two principal evidence classes. Serological assays—modified agglutination test (MAT), direct agglutination test (DAT), enzyme-linked immunosorbent assay (ELISA), indirect fluorescent antibody test (IFAT), latex agglutination test (LAT), Sabin–Feldman dye test (SFDT), and immunoblotting—were interpreted as evidence of previous or current exposure. Polymerase chain reaction (PCR), immunohistochemistry (IHC), parasite isolation, and bioassay were considered direct or tissue-based evidence. Seropositivity and direct-detection frequency were not treated as equivalent estimates of infection prevalence because antibody persistence, tissue distribution, sample matrix, and analytical sensitivity differ substantially [2,12].

### 2.3. Scope of Inference

The host comparison was ecological rather than phylogenetically exhaustive. The term “wild canids” is used throughout because the carnivore evidence synthesized here is restricted to red foxes, raccoon dogs, and wolves. The review emphasizes patterns that recur across studies but avoids causal attribution when transmission routes were not directly demonstrated. In particular, high seropositivity in a predator is compatible with trophic transmission but cannot exclude environmental oocyst exposure. Likewise, seropositivity in a cervid identifies exposure but does not reveal the contamination source, time of infection, viability of tissue cysts, or risk associated with a particular carcass. The synthesis was intentionally restricted to the five cervid and three canid species named above to maintain a tractable ecological comparison based on extractable prevalence records from free-ranging populations; other cervid and canid species were outside the prespecified scope.

### 2.4. Figure Preparation and Generative Artificial Intelligence Disclosure

Figure 1 was generated using ChatGPT (GPT-5, OpenAI; accessed on 9 July 2026) from scientific content and layout specifications defined by the authors. The authors critically reviewed, revised, and finalized the illustration for scientific accuracy and take full responsibility for the final figure.

## 3. Ecological Framework for Transmission

### 3.1. Environmental Oocyst Pathway

Oocysts shed by domestic or wild felids can sporulate in the environment and persist under favorable moisture and temperature conditions. They may be transported locally with soil particles or more widely through surface runoff and water flow. Vegetation contaminated by splash, dust, soil contact, or irrigation can expose browsing and grazing herbivores. Water sources can connect otherwise separated habitats, and environmental transport can decouple the location of infection from the location of oocyst shedding [3,13].

Cervids are useful within this oocyst transmission pathway because their herbivorous diet makes environmental exposure the most likely source of infection. Nevertheless, cervid seropositivity should not be interpreted as a direct measure of local oocyst density. Individual movement, home-range size, age, seasonal foraging, soil ingestion, water use, and contact with agricultural or peri-urban habitats can all modify cumulative exposure. Sentinel interpretation is strongest when host data are paired with spatially explicit information on land cover, precipitation, drainage, domestic-cat density, and wild-felid occurrence.

### 3.2. Trophic Pathway and Food-Web Integration

Intermediate hosts retain tissue cysts after infection, allowing transmission to predators and scavengers. A canid consuming rodents, lagomorphs, birds, ungulate tissues, placentas, or carrion may receive repeated infectious doses across its lifetime. This route is layered on top of environmental exposure from water, soil, contaminated prey surfaces, and anthropogenic food. Accordingly, canid infection can represent the integrated output of multiple prey species, habitats, and seasons. Global mammalian evidence that carnivorous dietary niches carry higher infection risk is consistent with this interpretation [4].

The contrast between cervids and canids is therefore not a simple distinction between “environmental” and “trophic” hosts. Both groups can ingest oocysts, but only canids routinely add tissue-cyst exposure through predation and scavenging. Figure 2 summarizes these complementary ecological pathways, which provide the conceptual basis for the surveillance framework discussed in later sections.

## 4. Evidence in Wild Cervids

Evidence of *T. gondii* exposure has been documented in roe deer, red deer, and sika deer across Europe and Asia, in white-tailed deer in North America and Europe, and in water deer in South Korea. Table 1 separates antibody-based evidence from direct detection. The wide ranges should be interpreted as study-specific results rather than species-level global prevalence estimates. Differences in assay cut-off, sample origin, age distribution, hunting or rescue context, geography, and sample size preclude a valid unadjusted ranking of species.

### 4.1. Roe Deer

Available data on *T. gondii* infection in roe deer (*Capreolus capreolus*) in Asia remain limited. Antibodies were detected in one of six rescued roe deer in Korea, whereas a small survey in China detected no seropositive animals [14,15], which cannot support robust geographic comparison. Direct detection has also been inconsistent: PCR identified *T. gondii* DNA in 3 of 121 roe deer in one Italian survey but not in another Italian dataset or the Korean sample [8,15,24]. These findings illustrate why a positive antibody test and a negative PCR result are not contradictory; chronic infection may produce durable antibodies while tissue cysts remain sparse and unevenly distributed.

European roe deer have been studied much more extensively, primarily through hunting programs. Reported seropositivity ranged from 12.8% in Germany to 60.0% in France, with intermediate estimates from Finland, Poland, Denmark, Slovenia, and Spain [16,17,18,19,20,21,22,23]. The magnitude of this range is unlikely to reflect host susceptibility alone. Regional oocyst pressure, sample-age composition, habitat moisture, felid density, and assay characteristics are plausible contributors.

### 4.2. Red Deer

Red deer (*Cervus elaphus*) are broadly distributed in Europe and parts of Asia and are important game animals. Serological estimates ranged from 6.4% in Germany to 30.7% in Spain, with evidence of exposure in Poland, Denmark, France, Slovenia, and China [14,16,20,21,22,23,25]. Spanish studies alone produced substantially different results across populations and years, emphasizing the influence of local ecology and study design [18,19,25].

PCR did not detect parasite DNA in the small Italian red deer sample included in the evidence table [8]. That result should not be used to infer absence from the population because only a limited quantity of selected tissue can be examined, and chronic tissue cysts are heterogeneously distributed. For surveillance, serology provides the more efficient population-exposure measure, whereas tissue-based methods are most informative when standardized tissue masses, multiple predilection sites, and sensitive targets are used.

### 4.3. Sika Deer

Sika deer (*Cervus nippon*) are native to East Asia and introduced in several European countries. Available studies are fewer and highly heterogeneous. Seropositivity was 7.1% in a small Danish sample and 1.9% in a Japanese LAT study, whereas a Japanese SFDT survey reported 47.5% [21,26,27]. The contrast may represent geographic and temporal variation, but assay and cut-off differences are equally important. It should therefore not be presented as evidence of a temporal increase or intrinsic population difference without direct methodological comparison.

### 4.4. White-Tailed Deer

White-tailed deer (*Odocoileus virginianus*) are among the most extensively studied wild cervids. Surveys in the United States frequently reported seropositivity between approximately 41% and 62%, including studies from Pennsylvania, Ohio, Illinois, New York, and the southeastern United States [28,29,30,31,32,33,34]. Estimates from Finland and northern Mexico were lower [17,35]. These data support widespread exposure but also demonstrate that a species-level average would obscure meaningful spatial variation.

White-tailed deer are epidemiologically important because they connect environmental contamination with human exposure through hunting and venison consumption. Deer are presumed to acquire infection predominantly by ingesting oocysts, after which tissue cysts may persist in edible tissues. A documented outbreak among Canadian hunters was associated with consumption of undercooked deer meat harvested in the United States, demonstrating that wildlife surveillance has direct food-safety relevance rather than merely ecological interest [9].

### 4.5. Water Deer

The water deer (*Hydropotes inermis*) is native to East Asia and is especially abundant in the Republic of Korea. An early study of rescued wildlife detected antibodies in 4 of 37 animals and *T. gondii* DNA in 1 of 37, whereas a nationwide survey later detected antibodies in 135 of 452 Korean water deer [15,36]. The larger dataset confirms that exposure is geographically widespread enough to justify systematic surveillance, while the contrast between antibody and PCR results again reflects the different endpoints of the assays.

Water deer have particular surveillance value in Korea because they occur in agricultural, riparian, peri-urban, and forest-edge environments and are frequently encountered through roadkill and wildlife-rescue systems. These passive-sampling networks can support repeated, spatially explicit monitoring with lower field-capture costs. However, rescue and roadkill samples may overrepresent animals from developed landscapes or animals in poor condition and should not automatically be treated as random population samples.

### 4.6. Cervid Synthesis

Across cervids, the most defensible common interpretation is that exposure is widespread but spatially heterogeneous. Reported seropositivity varies substantially both within and among species. However, these differences should be interpreted cautiously because relatively few studies were designed for direct comparison across geographic regions or host species. Variation in diagnostic methods, sampled age classes, tissue or sample types, and study design probably contributed to the observed heterogeneity, limiting direct comparisons among studies. Consequently, the available evidence is more robust for identifying broad ecological patterns and potential environmental hotspots than for ranking infection risk among regions or host species. The ecological signal is strongest when serological data are compared among age-stratified animals sampled using the same diagnostic assay within defined landscapes or time periods and interpreted alongside local felid and hydrological information.

## 5. Evidence in Wild Canids

Evidence of *T. gondii* exposure has been documented in red foxes in Europe and North America, in raccoon dogs in Europe and Asia, and in wolves in Europe and North America. Table 2 summarizes red foxes, raccoon dogs, and wolves. As in cervids, serology and direct detection are separated. The generally higher reported seropositivity in many canid studies is ecologically plausible, but unadjusted comparisons with cervids remain vulnerable to confounding by age structure, sampling source, test performance, and geography.

### 5.1. Red Fox

The red fox (*Vulpes vulpes*) is a dietary generalist that consumes small mammals, birds, invertebrates, carrion, fruit, and anthropogenic food. This breadth creates multiple opportunities for infection. Serological surveys in France, Poland, Spain, Hungary, Germany, Italy, Austria, and Portugal have often reported moderate-to-high exposure, although estimates varied widely and some were based on very small samples [22,39,41,42,45,48,52,53]. North American estimates ranged from no positives in one small Alaskan sample to high positivity in other small datasets [28,29,38]. The extreme 0% and 100% values from samples of fewer than ten animals demonstrate the instability of raw percentages at low sample sizes.

PCR-based detection ranged from low values in Czechia and the United Kingdom to higher values in Serbia, Italy, Belgium, Poland, and coastal California [8,12,37,39,43,44,47,49,51]. Immunohistochemistry in a German pathological survey detected no positive foxes [46]. These direct-detection results are strongly conditioned by tissue choice, sample mass, preservation, and assay target and should not be expected to reproduce serological estimates.

Red foxes are promising food-web sentinels because they are abundant, occupy rural and urbanizing landscapes, and consume prey from several trophic levels. Their use is particularly informative when stomach-content, stable-isotope, GPS, or land-use data are available. Without such ecological metadata, a high antibody frequency indicates cumulative exposure but does not identify the relative contribution of predation, scavenging, water, or soil.

### 5.2. Raccoon Dog

The raccoon dog (*Nyctereutes procyonoides*) is an omnivorous canid with a diet that can include small vertebrates, carrion, amphibians, invertebrates, fruit, and other plant material. This ecological flexibility creates both trophic and environmental exposure, but the proportion of vertebrate prey may be lower or more seasonal than in red foxes [11]. Reported antibody frequencies ranged from 4.3% in a small Korean rescue sample to 42.7% in Denmark, with intermediate values in Poland and no positives in a five-animal Russian sample [15,39,54,56,57].

PCR detected low frequencies in Polish samples and no positives in very small samples from Slovakia, Czechia, or Korea [15,39,40,51,55,58]. A German IHC survey was also negative [46]. The Korean fecal PCR study should be interpreted particularly cautiously because fecal detection addresses a different matrix from brain or muscle and may be affected by low shedding, environmental degradation, and the fact that canids are intermediate rather than definitive hosts.

Raccoon dogs may be especially useful at the rural–urban interface, where scavenging, roadkill consumption, agricultural habitats, and human food subsidies overlap. Their surveillance potential in East Asia is substantial, but current Korean sample sizes are insufficient for robust regional inference. A coordinated rescue-center and roadkill program could overcome this limitation if the sample origin and carcass condition are standardized.

### 5.3. Wolf

Wolves (*Canis lupus*) are apex or high-level predators whose exposure is expected to be strongly influenced by repeated consumption of infected ungulates and other vertebrate prey. Serological evidence has been reported in Spain, Italy, Poland, and multiple United States populations, with estimates ranging from approximately 9% to 47.6% [37,41,59,60,61,62,63]. PCR detected parasite DNA in wolves from Serbia and Italy but not in the single animal tested in Slovakia [38,47,55].

Wolves can integrate infection across large home ranges and diverse prey populations, but this strength complicates spatial attribution. A seropositive wolf may have acquired infection far from its sampling location. Furthermore, wolf samples are often opportunistic and derived from carcasses, management removals, or long-term ecological projects, creating demographic and geographic selection. Surveillance designs should therefore link infection data to movement history and diet whenever possible.

An ecological study in Yellowstone wolves reported an association between *T. gondii* seropositivity and risk-taking behaviors, including increased likelihood of dispersal and pack leadership [61]. This observation is scientifically important but should not be generalized as proof of parasite-driven behavioral manipulation in all wild canids. Observational associations may be influenced by age, habitat use, cougar exposure, or other correlated variables, and mechanistic inference requires longitudinal and experimental support.

### 5.4. Canid Synthesis

Canid studies collectively support broad exposure to *T. gondii*, particularly in red foxes and wolves. However, the evidence is not sufficient to assign a universal ranking among canid species. Red foxes have the largest and most geographically diverse evidence base, raccoon-dog data remain patchy, and wolf estimates often arise from specialized or small samples.

Compared with cervids, interpretation of canid seroprevalence is further complicated by the diversity of potential exposure pathways. Predation, scavenging, and environmental contamination may all contribute to infection, but their relative importance cannot be resolved from observational prevalence studies alone. Together with variation in study design and diagnostic methodology, this limits direct comparisons among geographic regions and species. Consequently, the current evidence is more informative for identifying broad ecological trends than for quantifying the contribution of individual transmission pathways.

## 6. Feeding Ecology and Transmission Dynamics

### 6.1. Environmental Exposure in Browsers and Grazers

Cervid feeding brings the muzzle repeatedly into contact with vegetation, soil particles, and surface water. Browsing height, snow cover, drought, supplemental feeding, and use of riparian habitats can alter the probability of ingesting oocysts. Environmental exposure may also be concentrated around farms, settlements, refuse sites, or feeding stations where domestic cats and prey species aggregate. Thus, “herbivory” does not represent a uniform exposure class; it interacts with habitat and management.

Within cervids, feeding-niche differentiation may further modulate exposure. Browsers such as roe deer feed selectively on shrubs and forbs and may encounter oocysts mainly through soil splash, low-shrub contact, and mineral licking, whereas grazers and intermediate feeders such as white-tailed deer and water deer forage closer to the soil–vegetation interface and in damp riparian or agricultural margins where oocysts can accumulate after runoff. This distinction is offered as an ecological hypothesis to be tested with paired diet and exposure data rather than as a quantitative determinant of seropositivity, because forage height, habitat moisture, and felid contamination are not independent in real landscapes.

### 6.2. Trophic Amplification in Predators and Scavengers

Canids add a trophic pathway because tissue cysts can be consumed with prey and carrion. Small mammals are especially plausible bridge hosts because they can acquire oocysts environmentally and are frequently eaten by foxes and other canids. Ungulate carcasses and offal may provide additional exposure to wolves, foxes, and raccoon dogs. Repeated predation can create a cumulative dose opportunity that is not available to strict herbivores. This mechanism is consistent with sympatric studies in which carnivores showed higher reported positivity than herbivores sampled from the same region [8,44].

Nevertheless, trophic amplification should be described as a supported hypothesis rather than a route proven by serology. Direct demonstration would require linking parasite genotypes among felids, environmental matrices, prey, and predators, or combining dietary reconstruction with infection status. Genotyping studies in wildlife show that canids can harbor lineages circulating within sylvatic systems, but genotype matching at the local food-web scale remains uncommon [43,47,60].

### 6.3. Age, Sex, and Movement

Age is among the most consistent host-level correlates because exposure accumulates over time. This pattern has also been observed in raccoon dogs and other wild hosts [54,56]. By contrast, sex effects are inconsistent. Many studies report no significant association, whereas isolated differences may reflect sex-specific movement, dispersal, reproductive behavior, or habitat use rather than biological susceptibility. Because males and females can be sampled differently by hunting, trapping, or rescue programs, sex effects should be evaluated together with age and sampling method.

Movement can increase the number of contaminated habitats encountered and may explain part of the association between vagility and infection at the global scale [4]. This has different implications for the two host groups. A cervid with a modest home range may provide relatively local environmental information, whereas a dispersing wolf may integrate exposure over hundreds of kilometers. Surveillance interpretation should therefore match the spatial scale of the host’s ecology.

### 6.4. Climate, Hydrology, and Anthropogenic Modification

Oocyst survival and transport are favored by moisture and moderated by temperature. The multilayered oocyst wall confers substantial resistance to mechanical and chemical stress, but viability is reduced by desiccation, ultraviolet radiation, and high soil-surface temperatures. As a result, local outcomes depend on shade, soil type, hydrological connectivity, and microclimate. Rainfall and snowmelt can disperse oocysts from terrestrial sources into streams and floodplains, concentrating contamination along riparian foraging zones, while drought may concentrate animals at limited water points even when long-term oocyst survival is reduced. Seasonal serological patterns are consequently difficult to interpret because antibodies integrate prior exposure rather than only current-season contamination [3].

Human land use modifies transmission by changing felid abundance, prey communities, food subsidies, and water flow. Free-roaming domestic cats can create high oocyst pressure near farms and settlements, while refuse and roadkill can concentrate scavengers. Suburban white-tailed deer and foxes illustrate how wildlife infection can emerge from coupled natural and anthropogenic cycles [32,33,45]. A robust One Health interpretation therefore requires data on domestic animals and landscape management rather than treating wildlife as an isolated reservoir.

## 7. Surveillance and One Health Implications

### 7.1. Cervids as Environmental Sentinels

Cervids are attractive sentinel hosts because they are widely distributed, often legally harvested, and readily sampled through hunting, roadkill, and rescue networks. Their herbivorous diet provides a relatively direct link to environmental oocyst exposure. However, cervid surveillance should be interpreted as evidence of environmental exposure rather than proof of a specific contamination source, definitive host, or environmental matrix.

### 7.2. Canids as Food-Web Integrators

Canids can complement cervid surveillance by indicating whether infection is circulating through prey communities and scavenging pathways. Red foxes are suitable for broad geographic monitoring because they are abundant and frequently collected through hunting or roadkill. Wolves offer integration across large prey networks but are less available and less spatially specific. Raccoon dogs may be particularly informative in wetland, agricultural, and peri-urban mosaics where dietary omnivory and scavenging connect multiple transmission sources.

The most informative design is paired surveillance. Cervid seropositivity with low canid exposure might suggest environmental contamination without extensive trophic amplification, whereas high values in both groups would be compatible with widespread oocyst exposure and food-web circulation. Such interpretations remain hypotheses until adjusted for assay, age, and sampling differences, but paired host guilds provide more epidemiological information than either group alone.

### 7.3. Venison, Hunters, and Food Safety

Cervid infection creates a pathway from environmental contamination to human food exposure, but the consumer hazard depends specifically on whether viable tissue cysts are present in edible tissues rather than on infection markers alone. The principal risk arises from the consumption of raw or undercooked meat, while hunters may also be exposed during field dressing and handling of carcasses, organs, and raw meat. The Quebec outbreak among deer hunters demonstrates that clinically apparent clusters can occur after consumption of undercooked venison [9].

However, surveillance findings should be interpreted cautiously, because seropositivity indicates exposure but does not demonstrate that a carcass contains viable cysts, and molecular detection of parasite DNA does not by itself establish infectivity. Current approaches to assessing consumer risk therefore rely on direct detection in relevant tissues together with viability-based methods. Mouse or cat bioassays remain the reference standard for demonstrating viability, but these assays are costly, time-consuming, and ethically constrained, which limits their use in routine carcass-level assessment. In vitro approaches, particularly cell culture-based systems, are being developed as alternatives and may help determine whether parasites remain capable of multiplication after processing, but these methods are not yet sufficiently standardized for routine food-safety application in complex meat matrices. Experimental evidence further indicates that partial curing or salting may reduce infectivity without ensuring complete inactivation, underscoring the need to distinguish parasite detection from parasite viability when interpreting foodborne risk [64]. For this reason, quantitative assessment of consumer risk requires standardized tissue selection, direct detection, viability testing, and validation of processing and preparation methods. Until such approaches are more fully validated, a proportionate public-health response includes clear guidance on adequate cooking, hygienic carcass handling, avoidance of raw tasting, and cautious interpretation of serological surveillance as a direct measure of foodborne risk.

### 7.4. East Asian and Korean Surveillance Opportunities

East Asian evidence remains less extensive than European and North American evidence, despite dense interfaces among wildlife, domestic cats, agriculture, and urban areas. Korean water deer and raccoon dogs form an ecologically useful pair: water deer represent environmental exposure in a highly abundant herbivore that uses riparian and agricultural margins, whereas raccoon dogs integrate scavenging, omnivory, and peri-urban habitat use. Existing Korean studies establish feasibility but are uneven in sample size and matrix [15,36,58], and the recent nationwide water-deer survey has substantially raised the baseline for what is now possible in regional surveillance [36].

A national or regional program could use wildlife rescue centers, roadkill collection, and disease surveillance facilities to obtain standardized serum, heart, brain, and skeletal-muscle samples. Linking these samples to land cover, watershed, road density, domestic-cat proxies, season, age, and body condition would create a high-value One Health dataset. Repeated surveillance could also evaluate whether urban expansion, climate variability, or wildlife management changes alter transmission over time. Such an approach would simultaneously address ecological, food-safety, and zoonotic questions within a single sampling architecture.

## 8. Diagnostic and Interpretive Challenges

### 8.1. Serology Does Not Equal Active or Viable Infection

Antibodies indicate host exposure and may persist long after initial infection. They do not specify when or where infection occurred, whether viable cysts remain in a tested tissue, or which route caused infection. Species-specific test validation is limited for many wildlife hosts, and cut-off values may be adapted from domestic animals. Cross-reaction, matrix effects, and differences in immunoglobulin detection can alter apparent prevalence [12,65].

Conversely, a negative PCR result does not exclude infection. Tissue cysts are focal, parasite burden may be low, and small samples may miss infected areas. DNA degradation during carcass decomposition, repeated freezing and thawing, or delayed processing further reduces sensitivity. Direct-detection estimates therefore depend on tissue type, mass, homogenization, target copy number, extraction method, inhibition control, and replication strategy [2,66].

### 8.2. Assay Heterogeneity

The reviewed studies used MAT, DAT, ELISA, IFAT, LAT, SFDT, immunoblotting, PCR, and IHC. Even within one assay family, antigen preparation, dilution threshold, conjugate, and positivity rule differed. A comparison of DAT, ELISA, and PCR in wild animals demonstrated only moderate agreement between antibody methods and substantially fewer PCR-positive samples than seropositive samples [39]. Therefore, prevalence differences across methods should not be interpreted as biological differences without method calibration.

Future wildlife studies should report assay manufacturer or antigen source, cut-off rationale, validation host, controls, sample matrix, storage conditions, tissue mass, genetic target, replication, and handling of equivocal results. Where possible, a subset should be tested with two complementary methods. This would allow estimates of agreement and help distinguish assay effects from ecological effects.

### 8.3. Sampling and Denominator Bias

Hunted animals, road-killed animals, rescued wildlife, and found-dead carcasses represent different source populations. Hunting samples may be selected by age, sex, season, and location; roadkill may overrepresent mobile animals in fragmented landscapes; rescue samples may overrepresent illness, urban contact, or juvenile age; and carcass studies may be limited by autolysis. These processes affect both the numerator and denominator of reported positivity.

Small samples deserve particular caution. Percentages such as 0% or 100% from one to six animals are mathematically correct but epidemiologically unstable. Reporting exact confidence intervals and retaining numerator/denominator information prevents overinterpretation. Comparative analyses should also avoid treating multiple assays on the same animals as independent population estimates.

### 8.4. Limits of Causal Inference

The central ecological interpretation of this review that canids have additional trophic exposure compared with cervids is biologically well supported, but most individual studies are cross-sectional. Serology cannot attribute the route, and geographic comparisons combine many uncontrolled variables. Statements that feeding ecology “explains” prevalence should therefore be replaced with language indicating consistency, plausibility, or association. Stronger causal evidence will require paired prey–predator sampling, genotype or genomic linkage, diet reconstruction, environmental sampling, and longitudinal designs.

## 9. Priorities for Future Research

Five priorities would substantially improve inference. First, harmonized surveillance should distinguish exposure from direct detection and report complete diagnostic metadata. Second, studies should collect age, sex, body condition, precise location, season, and sampling source as minimum ecological variables. Third, cervids and canids should be sampled within the same landscapes and time periods so that host-guild comparisons are not confounded by region. Fourth, direct-detection studies should standardize tissue selection and include sufficiently large, homogenized samples from more than one predilection site. Fifth, infection data should be linked to felid activity, hydrology, land use, prey composition, and human food practices.

Quantitative synthesis may become appropriate when these reporting standards are met. A future meta-analysis should stratify by host species, assay class, cut-off, sample matrix, age, continent, and sampling source rather than pooling all records. Hierarchical models could then estimate study-level and species-level effects while accounting for non-independence. Until then, structured narrative comparison is more defensible than a single pooled prevalence.

Molecular epidemiology is another priority. Genotyping or whole-genome approaches could test whether identical or related parasite lineages occur in local felids, cervids, prey, and canids. Such work would move the field from inference based on trophic position toward direct reconstruction of transmission networks. In parallel, game-meat studies should evaluate tissue distribution and viability to translate wildlife infection evidence into realistic consumer-risk estimates.

## 10. Conclusions

Wild cervids and wild canids occupy complementary positions in the terrestrial ecology of *Toxoplasma gondii*. Cervids primarily reflect environmental exposure, whereas canids integrate both environmental and trophic pathways. Accordingly, higher reported seropositivity is frequently observed in canids; however, this pattern should be interpreted cautiously because the available evidence is derived largely from observational studies with substantial methodological heterogeneity, including variation in diagnostic assays, sampling source, age structure, and geographic context. Although these observations are compatible with differences in ecological exposure opportunities, they do not demonstrate a causal relationship between feeding ecology and infection risk.

The principal practical implication is not to prioritize one host guild over the other, but to interpret them as complementary wildlife sentinels. Paired cervid–canid surveillance, supported by standardized serological testing, targeted tissue detection, ecological and environmental metadata, and appropriate food-safety assessment, can provide a more comprehensive understanding of environmental contamination and food-web transmission. Because the ecological roles of these host groups are broadly conserved across terrestrial ecosystems, this complementary framework can be adapted to different ecological and geographic contexts, thereby strengthening wildlife disease surveillance and One Health risk assessment.

Future research should prioritize longitudinal studies, greater standardization of diagnostic approaches, and the integration of environmental, molecular, and genomic data to better reconstruct transmission pathways and distinguish temporal from spatial variation in parasite circulation. Korean water deer and raccoon dogs provide a particularly valuable East Asian model for implementing this integrated surveillance framework, complementing the more extensive evidence available from Europe and North America. Together, these advances will strengthen epidemiological surveillance, improve ecosystem health monitoring, and support evidence-based strategies for reducing foodborne and environmental exposure to *T. gondii*.

## Figures and Tables

**Table 1:** Reported detection of *Toxoplasma gondii* in free-ranging wild cervids. Serological and direct-detection records are displayed separately by evidence category.

Host	Continent	Country	Assay	Evidence	Positive/Tested	Reported Result	Ref.
Roe deer (*Capreolus capreolus*)	Asia	China	ELISA	Exposure	0/16	0%	[14]
		South Korea	ELISA	Exposure	1/6	16.70%	[15]
		South Korea	PCR	Direct detection	0/6	0%	[15]
	Europe	Poland	ELISA	Exposure	28/92	30.40%	[16]
		Finland	DAT	Exposure	3/17	17.60%	[17]
		Spain	MAT	Exposure	3/22	13.60%	[18]
		Spain	MAT	Exposure	141/356	39.60%	[19]
		Germany	ELISA	Exposure	16/125	12.80%	[20]
		Denmark	ELISA	Exposure	18/55	32.70%	[21]
		France	MAT	Exposure	36/60	60%	[22]
		Slovenia	ELISA	Exposure	62/134	46.27%	[23]
		Italy	PCR	Direct detection	3/121	2.48%	[8]
		Italy	PCR	Direct detection	0/72	0%	[24]
Red deer (*Cervus elaphus*)	Europe	Italy	PCR	Direct detection	0/13	0%	[8]
		Poland	ELISA	Exposure	133/552	24.10%	[16]
		Spain	MAT	Exposure	112/1063	10.50%	[18]
		Spain	MAT	Exposure	130/423	30.70%	[25]
		Spain	MAT	Exposure	92/553	16.60%	[19]
		Germany	ELISA	Exposure	3/47	6.40%	[20]
		Denmark	ELISA	Exposure	74/272	27.20%	[21]
		France	MAT	Exposure	4/24	17%	[22]
		Slovenia	ELISA	Exposure	32/113	28.32%	[23]
	Asia	China	ELISA	Exposure	6/56	10.70%	[14]
Sika deer (*Cervus nippon*)	Europe	Denmark	ELISA	Exposure	1/14	7.10%	[23]
	Asia	Japan	LAT	Exposure	2/107	1.90%	[26]
		Japan	SFDT	Exposure	38/80	47.50%	[27]
White-tailed deer (*Odocoileus virginianus*)	Europe	Finland	DAT	Exposure	36/135	26.70%	[17]
	North America	USA	MAT	Exposure	34/73	46.50%	[28]
		USA	MAT	Exposure	49/79	62%	[29]
		USA	ELISA	Exposure	71/144	49.31%	[30]
		USA	MAT	Exposure	99/241	41%	[31]
		USA	MAT	Exposure	261/444	58.80%	[32]
		USA	MAT	Exposure	248/443	55.90%	[33]
		USA	ELISA	Exposure	113/268	42%	[34]
		Mexico	ELISA	Exposure	74/532	13.90%	[35]
Water deer (*Hydropotes inermis*)	Asia	South Korea	ELISA	Exposure	4/37	10.80%	[15]
		South Korea	PCR	Direct detection	1/37	2.70%	[15]
		South Korea	ELISA	Exposure	135/452	29.90%	[36]

Abbreviations: DAT, direct agglutination test; ELISA, enzyme-linked immunosorbent assay; LAT, latex agglutination test; MAT, modified agglutination test; PCR, polymerase chain reaction; SFDT, Sabin–Feldman dye test. Estimates based on very small samples should be interpreted descriptively. Antibody detection indicates exposure and is not directly equivalent to tissue-based detection.

**Table 2:** Reported detection of *Toxoplasma gondii* in free-ranging wild canids. Serological and direct-detection records are displayed separately by evidence category.

Host	Continent	Country	Assay	Evidence	Positive/Tested	Reported Result	Ref.
Red fox (*Vulpes* *vulpes*)	North America	USA	MAT	Exposure	1/1	100%	[28]
		USA	MAT	Exposure	17/20	85%	[29]
		USA	MAT	Exposure	0/9	0%	[37]
		USA	PCR	Direct detection	3/11	27%	[13]
	Europe	Italy	PCR	Direct detection	15/94	15.96%	[8]
		Italy	PCR	Direct detection	17/71	23.9%	[38]
		France	MAT	Exposure	14/19	73.7%	[22]
		Poland	PCR	Direct detection	20/102	19.6%	[39]
		Poland	DAT/ELISA	Exposure	79/102	77.5%	[39]
		Poland	PCR	Direct detection	11/148	7.4%	[40]
		Spain	MAT	Exposure	66/102	64.7%	[41]
		Portugal	MAT	Exposure	6/6	100%	[42]
		UK	PCR	Direct detection	5/83	6%	[43]
		Belgium	PCR	Direct detection	57/304	18.8%	[44]
		Germany	Immunoblot	Exposure	301/380	79.2%	[45]
		Germany	IHC	Direct detection	0/79	0%	[46]
		Serbia	PCR	Direct detection	10/28	35.7%	[47]
		Hungary	DAT	Exposure	228/337	68%	[48]
		Czechia	PCR	Direct detection	10/100	10%	[49]
		Czechia	IFAT	Exposure	3/80	3.8%	[50]
		Czechia	PCR	Direct detection	2/152	1.32%	[51]
		Austria	IFAT	Exposure	33/94	35%	[52]
		Italy	IFAT	Exposure	79/120	65.8%	[53]
Raccoon dog (*Nyctereutes procyonoides*)	Europe	Poland	MAT	Exposure	25/89	28.09%	[54]
		Poland	PCR	Direct detection	1/12	8.3%	[39]
		Poland	DAT	Exposure	3/12	25%	[39]
		Poland	PCR	Direct detection	1/13	7.7%	[40]
		Slovakia	PCR	Direct detection	0/1	0%	[55]
		Czechia	PCR	Direct detection	0/3	0%	[51]
		Germany	IHC	Direct detection	0/10	0%	[46]
		Denmark	ELISA	Exposure	97/227	42.7%	[56]
	Asia	Russia*	ELISA	Exposure	0/5	0%	[57]
		South Korea	ELISA	Exposure	1/23	4.3%	[15]
		South Korea	PCR	Direct detection	0/23	0%	[15]
		South Korea	PCR	Direct detection	0/11	0%	[58]
Wolf (*Canis* *lupus*)	North America	USA	MAT	Exposure	11/125	9%	[59]
		USA	MAT	Exposure	29/324	9%	[38]
		USA	MAT	Exposure	50/105	47.6%	[60]
		USA	ELISA	Exposure	29/115	25.2%	[61]
	Europe	Spain	MAT	Exposure	15/32	46.9%	[41]
		Serbia	PCR	Direct detection	2/10	20%	[47]
		Italy	IFAT	Exposure	34/128	26.6%	[62]
		Italy	PCR	Direct detection	4/14	28.6%	[37]
		Poland	ELISA	Exposure	4/19	21%	[63]
		Slovakia	PCR	Direct detection	0/1	0%	[55]

Abbreviations: DAT, direct agglutination test; ELISA, enzyme-linked immunosorbent assay; IFAT, indirect fluorescent antibody test; IHC, immunohistochemistry; MAT, modified agglutination test; PCR, polymerase chain reaction. Small-sample estimates, including 0% or 100% values, are descriptive and should not be interpreted as stable population parameters. * Study conducted in Lazovskii State Nature Reserve (Russian Far East, Asia).

## Data Availability

No new data were created or analyzed in this study. Data sharing is not applicable to this article.

## References

[1] Tenter A.M., Heckeroth A.R., Weiss L.M. (2000). *Toxoplasma gondii*: From animals to humans. Int. J. Parasitol..

[2] Robert-Gangneux F., Dardé M.-L. (2012). Epidemiology of and diagnostic strategies for toxoplasmosis. Clin. Microbiol. Rev..

[3] Shapiro K., Bahia-Oliveira L., Dixon B., Dumètre A., de Wit L.A., VanWormer E., Villena I. (2019). Environmental transmission of *Toxoplasma gondii*: Oocysts in water, soil and food. Food Waterborne Parasitol..

[4] Wilson A.G., Lapen D.R., Provencher J.F., Wilson S. (2024). The role of species ecology in predicting *Toxoplasma gondii* prevalence in wild and domesticated mammals globally. PLoS Pathog..

[5] Amouei A., Mizani A., Arabian M., Teshnizi S.H., Gevorgyan R., Amuei F., Dodangeh S., Sadeghi D., Naeimi S., Daryani A. (2025). Prevalence of toxoplasmosis in natural ungulates as human zoonotic meat-borne pathogens: A systematic review and meta-analysis. J. Food Sci..

[6] Chen C., Qin S.-Y., Yang X., Li X.-M., Cai Y., Lei C.-C., Zhao Q., Hany M.E., Cao H. (2024). Global prevalence and risk factors associated with *Toxoplasma gondii* infection in wild birds: A systematic review and meta-analysis. Prev. Vet. Med..

[7] Kutz S.J., Elkin B.T., Panayi D., Dubey J.P. (2001). Prevalence of *Toxoplasma gondii* antibodies in barren-ground caribou (*Rangifer tarandus groenlandicus*) from the Canadian Arctic. J. Parasitol..

[8] Ferroglio E., Bosio F., Trisciuoglio A., Zanet S. (2014). *Toxoplasma gondii* in sympatric wild herbivores and carnivores: Epidemiology of infection in the Western Alps. Parasites Vectors.

[9] Gaulin C., Ramsay D., Thivierge K., Tataryn J., Courville A., Martin C., Cunningham P., Désilets J., Morin D., Dion R. (2020). Acute toxoplasmosis among Canadian deer hunters associated with consumption of undercooked deer meat hunted in the United States. Emerg. Infect. Dis..

[10] Dubey J.P., Murata F.H.A., Cerqueira-Cézar C.K., Kwok O.C.H. (2021). Recent epidemiologic and clinical *Toxoplasma gondii* infections in wild canids and other carnivores: 2009–2020. Vet. Parasitol..

[11] Sutor A., Kauhala K., Ansorge H. (2010). Diet of the raccoon dog *Nyctereutes procyonoides*—A canid with an opportunistic foraging strategy. Acta Theriol..

[12] Liu Q., Wang Z.-D., Huang S.-Y., Zhu X.-Q. (2015). Diagnosis of toxoplasmosis and typing of *Toxoplasma gondii*. Parasites Vectors.

[13] Miller M.A., Miller W.A., Conrad P.A., James E.R., Melli A.C., Leutenegger C.M., Dabritz H.A., Packham A.E., Paradies D., Harris M. (2008). Type X *Toxoplasma gondii* in a wild mussel and terrestrial carnivores from coastal California: New linkages between terrestrial mammals, runoff and toxoplasmosis of sea otters. Int. J. Parasitol..

[14] Wu J.-Y., Li J.-J., Wang D.-F., Wei Y.-R., Meng X.-X., Tuerxun G., Bolati H., Liu K.-K., Muhan M., Shahan A. (2020). Seroprevalence of five zoonotic pathogens in wild ruminants in Xinjiang, Northwest China. Vector-Borne Zoonotic Dis..

[15] Hong S.-H., Kim H.-J., Jeong Y.-I., Cho S.-H., Lee W.-J., Kim J.-T., Lee S.-E. (2017). Serological and molecular detection of *Toxoplasma gondii* and *Babesia microti* in the blood of rescued wild animals in Gangwon-do (Province), Korea. Korean J. Parasitol..

[16] Witkowski L., Czopowicz M., Nagy D.A., Potarniche A.V., Aoanei M.A., Imomov N., Mickiewicz M., Welz M., Szaluś-Jordanow O., Kaba J. (2015). Seroprevalence of *Toxoplasma gondii* in wild boars, red deer and roe deer in Poland. Parasite.

[17] Jokelainen P., Näreaho A., Knaapi S., Oksanen A., Rikula U., Sukura A. (2010). *Toxoplasma gondii* in wild cervids and sheep in Finland: North–south gradient in seroprevalence. Vet. Parasitol..

[18] Almería S., Cabezón O., Paniagua J., Cano-Terriza D., Jiménez-Ruiz S., Arenas-Montes A., Dubey J.P., García-Bocanegra I. (2018). *Toxoplasma gondii* in sympatric domestic and wild ungulates in the Mediterranean ecosystem. Parasitol. Res..

[19] Castro-Scholten S., Cano-Terriza D., Jiménez-Ruiz S., Almería S., Risalde M.A., Vicente J., Acevedo P., Arnal M.C., Balseiro A., Gómez-Guillamón F. (2021). Seroepidemiology of *Toxoplasma gondii* in wild ruminants in Spain. Zoonoses Public Health.

[20] Bier N.S., Stollberg K., Mayer-Scholl A., Johne A., Nöckler K., Richter M. (2020). Seroprevalence of *Toxoplasma gondii* in wild boar and deer in Brandenburg, Germany. Zoonoses Public Health.

[21] Stensgaard A.S., Sengupta M.E., Chriél M., Nielsen S.T., Petersen H.H. (2022). Sero-prevalence and risk factors of *Toxoplasma gondii* infection in wild cervids in Denmark. Int. J. Parasitol. Parasites Wildl..

[22] Aubert D., Ajzenberg D., Richomme C., Gilot-Fromont E., Terrier M.E., de Gevigney C., Game Y., Maillard D., Gibert P., Dardé M.L. (2010). Molecular and biological characteristics of *Toxoplasma gondii* isolates from wildlife in France. Vet. Parasitol..

[23] Žele Vengušt D., Krt B., Blagus R., Vengušt G., Bandelj P. (2024). Seroprevalence of infectious pathogens of zoonotic and veterinary importance in wild ruminants from Slovenia. Front. Vet. Sci..

[24] Ebani V.V., Trebino C., Guardone L., Bertelloni F., Cagnoli G., Altomonte I., Vignola P., Bongi P., Mancianti F. (2022). Retrospective molecular survey on bacterial and protozoan abortive agents in roe deer (*Capreolus capreolus*) from central Italy. Animals.

[25] Barroso P., García-Bocanegra I., Acevedo P., Palencia P., Carro F., Jiménez-Ruiz S., Almería S., Dubey J.P., Cano-Terriza D., Vicente J. (2020). Long-term determinants of the seroprevalence of *Toxoplasma gondii* in a wild ungulate community. Animals.

[26] Matsumoto J., Kako Y., Morita Y., Kabeya H., Sakano C., Nagai A., Maruyama S., Nogami S. (2011). Seroprevalence of *Toxoplasma gondii* in wild boars (*Sus scrofa leucomystax*) and wild sika deer (*Cervus nippon*) in Gunma Prefecture, Japan. Parasitol. Int..

[27] Hoshina T., Fukumoto S., Aonuma H., Saiki E., Hori S., Kanuka H. (2019). Seroprevalence of *Toxoplasma gondii* in wild sika deer in Japan. Parasitol. Int..

[28] Dubey J.P., Graham D.H., De Young R.W., Dahl E., Eberhard M.L., Nace E.K., Won K., Bishop H., Punkosdy G., Sreekumar C. (2004). Molecular and biologic characteristics of *Toxoplasma gondii* isolates from wildlife in the United States. J. Parasitol..

[29] Dubey J.P., Van Why K., Verma S.K., Choudhary S., Kwok O.C.H., Khan A., Behinke M.S., Sibley L.D., Ferreira L.R., Oliveira S. (2014). Genotyping *Toxoplasma gondii* from wildlife in Pennsylvania and identification of natural recombinants virulent to mice. Vet. Parasitol..

[30] Ledgerwood E.D., Luscier J.D. (2025). Seroprevalence of *Toxoplasma gondii* in white-tailed deer (*Odocoileus virginianus*) in New York State. Pathogens.

[31] Gerhold R.W., Saraf P., Chapman A., Zou X., Hickling G., Stiver W.H., Houston A., Souza M., Su C. (2017). *Toxoplasma gondii* seroprevalence and genotype diversity in select wildlife species from the southeastern United States. Parasites Vectors.

[32] Ballash G.A., Dubey J.P., Kwok O.C.H., Shoben A.B., Robison T.L., Kraft T.J., Dennis P.M. (2015). Seroprevalence of *Toxoplasma gondii* in white-tailed deer (*Odocoileus virginianus*) and free-roaming cats (*Felis catus*) across a suburban to urban gradient in northeastern Ohio. EcoHealth.

[33] Hollis-Etter K.M., Anchor C.L., Chelsvig J.E., Dubey J.P., Warner R.E. (2019). Suburban white-tailed deer seropositive for *Toxoplasma gondii* from Chicago, Illinois. Parasitol. Res..

[34] Schaefer J.J., Kirchgessner M.S., Whipps C.M., Mohammed H.O., Bunting E.M., Wade S.E. (2013). Prevalence of antibodies to *Toxoplasma gondii* in white-tailed deer (*Odocoileus virginianus*) in New York State, USA. J. Wildl. Dis..

[35] Olamendi-Portugal M., Caballero-Ortega H., Correa D., Sánchez-Alemán M.A., Cruz-Vázquez C., Medina-Esparza L., Ortega-S. J.A., Cantu A., García-Vázquez Z. (2012). Serosurvey of antibodies against *Toxoplasma gondii* and *Neospora caninum* in white-tailed deer from Northern Mexico. Vet. Parasitol..

[36] Hwang J., Kim J., Son K., Kim Y., Jeong H., Jheong W. (2024). Seroprevalence of *Toxoplasma gondii* infection in wild boar (*Sus scrofa*) and Korean water deer (*Hydropotes inermis argyropus*) in the Republic of Korea. Animals.

[37] Stieve E., Beckmen K., Kania S.A., Widner A., Patton S. (2010). *Neospora caninum* and *Toxoplasma gondii* antibody prevalence in Alaska wildlife. J. Wildl. Dis..

[38] Dakroub H., Sgroi G., D’Alessio N., Russo D., Serra F., Veneziano V., Rea S., Pucciarelli A., Lucibelli M.G., De Carlo E. (2023). Molecular survey of *Toxoplasma gondii* in wild mammals of Southern Italy. Pathogens.

[39] Kornacka A., Cybulska A., Bień J., Goździk K., Moskwa B. (2016). The usefulness of direct agglutination test, enzyme-linked immunosorbent assay and polymerase chain reaction for the detection of *Toxoplasma gondii* in wild animals. Vet. Parasitol..

[40] Sroka J., Karamon J., Wójcik-Fatla A., Dutkiewicz J., Bilska-Zając E., Zając V., Piotrowska W., Cencek T. (2019). *Toxoplasma gondii* infection in selected species of free-living animals in Poland. Ann. Agric. Environ. Med..

[41] Sobrino R., Cabezón O., Millán J., Pabón M., Arnal M.C., Luco D.F., Gortázar C., Dubey J.P., Almería S. (2007). Seroprevalence of *Toxoplasma gondii* antibodies in wild carnivores from Spain. Vet. Parasitol..

[42] Lopes A.P., Sargo R., Rodrigues M., Cardoso L. (2011). High seroprevalence of antibodies to *Toxoplasma gondii* in wild animals from Portugal. Parasitol. Res..

[43] Burrells A., Bartley P.M., Zimmer I.A., Roy S., Kitchener A.C., Meredith A., Wright S.E., Innes E.A., Katzer F. (2013). Evidence of the three main clonal *Toxoplasma gondii* lineages from wild mammalian carnivores in the UK. Parasitology.

[44] De Craeye S., Speybroeck N., Ajzenberg D., Dardé M.L., Collinet F., Tavernier P., Van Gucht S., Dorny P., Dierick K. (2011). *Toxoplasma gondii* and *Neospora caninum* in wildlife: Common parasites in Belgian foxes and Cervidae?. Vet. Parasitol..

[45] Herrmann D.C., Maksimov P., Maksimov A., Sutor A., Schwarz S., Jaschke W., Schliephake A., Denzin N., Conraths F.J., Schares G. (2012). *Toxoplasma gondii* in foxes and rodents from the German federal states of Brandenburg and Saxony-Anhalt: Seroprevalence and genotypes. Vet. Parasitol..

[46] Lempp C., Jungwirth N., Grilo M.L., Reckendorf A., Ulrich A., van Neer A., Bodewes R., Pfankuche V.M., Bauer C., Osterhaus A.D.M.E. (2017). Pathological findings in the red fox (*Vulpes vulpes*), stone marten (*Martes foina*) and raccoon dog (*Nyctereutes procyonoides*), with special emphasis on infectious and zoonotic agents in Northern Germany. PLoS ONE.

[47] Uzelac A., Klun I., Ćirović D., Penezić A., Ćirković V., Djurković-Djaković O. (2019). Detection and genotyping of *Toxoplasma gondii* in wild canids in Serbia. Parasitol. Int..

[48] Jakubek E.-B., Farkas R., Pálfi V., Mattsson J.G. (2007). Prevalence of antibodies against *Toxoplasma gondii* and *Neospora caninum* in Hungarian red foxes (*Vulpes vulpes*). Vet. Parasitol..

[49] Lukášová R., Marková J., Bártová E., Murat J.-B., Sedlák K. (2018). Molecular evidence of *Toxoplasma gondii*, *Neospora caninum*, and *Encephalitozoon cuniculi* in red foxes (*Vulpes vulpes*). J. Wildl. Dis..

[50] Bártová E., Slezáková R., Nágl I., Sedlák K. (2016). *Neospora caninum* and *Toxoplasma gondii* antibodies in red foxes (*Vulpes vulpes*) in the Czechia. Ann. Agric. Environ. Med..

[51] Hůrková L., Modrý D. (2006). PCR detection of *Neospora caninum*, *Toxoplasma gondii* and *Encephalitozoon cuniculi* in brains of wild carnivores. Vet. Parasitol..

[52] Wanha K., Edelhofer R., Gabler-Eduardo C., Prosl H. (2005). Prevalence of antibodies against *Neospora caninum* and *Toxoplasma gondii* in dogs and foxes in Austria. Vet. Parasitol..

[53] Brustenga L., Scarcelli S., Rigamonti G., Moretta I., Diaferia M., Morganti G., D’Avino N., Gobbi M., Ranucci A., Sgroi G. (2025). Seroprevalence of *Toxoplasma gondii*, *Neospora caninum* and *Encephalitozoon cuniculi* in red foxes (*Vulpes vulpes*) from Italy. Pathogens.

[54] Osten-Sacken N., Pikalo J., Steinbach P., Heddergott M. (2024). Prevalence of *Toxoplasma gondii* antibodies and risk factors in two sympatric invasive carnivores (*Procyon lotor* and *Nyctereutes procyonoides*) from Zgorzelec County, Poland. Pathogens.

[55] Turčeková L., Hurníková Z., Spišák F., Miterpáková M., Chovancová B. (2014). *Toxoplasma gondii* in protected wildlife in the Tatra National Park (TANAP), Slovakia. Ann. Agric. Environ. Med..

[56] Kjær L.J., Jensen L.M., Chriél M., Bødker R., Petersen H.H. (2021). The raccoon dog (*Nyctereutes procyonoides*) as a reservoir of zoonotic diseases in Denmark. Int. J. Parasitol. Parasites Wildl..

[57] Goncharuk M.S., Kerley L.L., Naidenko S.V., Rozhnov V.V. (2012). Prevalence of seropositivity to pathogens in small carnivores in adjacent areas of Lazovskii Reserve. Biol. Bull. Russ. Acad. Sci..

[58] Kumari P., Eo K.Y., Lee W.-S., Kimura J., Yamamoto N. (2021). DNA-based detection of *Leptospira wolffii*, *Giardia intestinalis* and *Toxoplasma gondii* in environmental feces of wild animals in Korea. J. Vet. Med. Sci..

[59] Zarnke R.L., Dubey J.P., Kwok O.C., Ver Hoef J.M. (2000). Serologic survey for *Toxoplasma gondii* in selected wildlife species from Alaska. J. Wildl. Dis..

[60] Dubey J.P., Velmurugan G.V., Rajendran C., Yabsley M.J., Thomas N.J., Beckmen K.B., Sinnett D., Ruid D., Hart J., Fair P.A. (2011). Genetic characterisation of *Toxoplasma gondii* in wildlife from North America revealed widespread and high prevalence of the fourth clonal type. Int. J. Parasitol..

[61] Meyer C.J., Cassidy K.A., Stahler E.E., Brandell E.E., Anton C.B., Stahler D.R., Smith D.W. (2022). Parasitic infection increases risk-taking in a social, intermediate host carnivore. Commun. Biol..

[62] Dini F.M., Musto C., De Nigris V.M., Bellinello E., Sampieri M., Merialdi G., Barca L., Delogu M., Galuppi R. (2024). Sero-epidemiological investigation on *Toxoplasma gondii* infection in Apennine wolf (*Canis lupus italicus*) and wild boar (*Sus scrofa*) in Italy. BMC Vet. Res..

[63] Goździk K., Mysłajek R.W., Kornacka-Stackonis A., Nowak S. (2026). Serological evidence of *Neospora caninum* and *Toxoplasma gondii* in gray wolves (*Canis lupus*) in Poland. Acta Parasitol..

[64] Opsteegh M., Dam-Deisz C., de Boer P., De Craeye S., Faré A., Hengeveld P., Luiten R., Schares G., van Solt-Smits C., Verhaegen B. (2020). Methods to assess the effect of meat processing on viability of *Toxoplasma gondii*: Towards replacement of mouse bioassay by in vitro testing. Int. J. Parasitol..

[65] Bokaba R.P., Dermauw V., Morar-Leather D., Dorny P., Neves L. (2022). *Toxoplasma gondii* in African wildlife: A systematic review. Pathogens.

[66] Rojas A., Germitsch N., Oren S., Sazmand A., Deak G. (2024). Wildlife parasitology: Sample collection and processing, diagnostic constraints, and methodological challenges in terrestrial carnivores. Parasites Vectors.

